# Perceived wintering latitude determines timing of song output in a migratory bird

**DOI:** 10.1002/ece3.5922

**Published:** 2019-12-28

**Authors:** Dustin E. Brewer, Clint A. McGill, Adam M. Fudickar

**Affiliations:** ^1^ Biosciences 2100 Central Michigan University Mount Pleasant MI USA; ^2^ Environmental Resilience Institute Indiana University Bloomington IN USA

**Keywords:** birdsong, connectivity, partial migration, photoperiod, timing

## Abstract

Migratory bird populations frequently consist of individuals that overwinter variable distances from the breeding site. Seasonal changes in photoperiod, which varies with latitude, underlie seasonal changes in singing frequency in birds. Therefore, migratory populations that consist of individuals that overwinter at different latitudes with large overwintering ranges could experience within‐population variation in seasonal production of song. To test the influence of overwintering latitude on intrapopulation variance in song production in the spring, we subjected two groups of Eastern Song Sparrows (*Melospiza melodia melodia*) from the same partially migratory breeding population to different photoperiodic schedules associated with a 1,300‐km difference in overwintering location. One group remained on the natural photoperiodic schedule of the breeding site (resident group) while the other group experienced a nonbreeding photoperiod that mimicked a southern migration in the fall followed by a northern migration back to the breeding site in the spring (migratory group). We compared song output between the two groups in three different stages (nonbreeding, prebreeding, and breeding). Little singing occurred during nonbreeding stage sample dates (20 November, 6 December) for the resident group, and no singing occurred for the migrant group. During the prebreeding stage (27 January, 7 February), significantly more singing occurred in the resident group than in the migrant group. During the breeding stage (21 March, 4 April), after a simulated migration for the migrants, song output was similar in both groups. These results suggest that within‐population variation in wintering latitude may contribute to variation in seasonal changes in singing behavior, which may covary with readiness to breed. Studies utilizing confirmed migrants and residents, rather than merely simulated migrants and residents, are also needed to better understand these processes.

## Graphical abstract

Migratory bird populations often consist of individuals that overwinter at different distances from a common breeding site. In the laboratory, we compared singing behavior between two groups of birds (one “migratory” and one “resident”) that we subjected to natural photoperiods of Tampa, Florida, and Bloomington, Indiana, respectively. We found that the residents sang more during the prebreeding stage, but after a simulated spring migration, song output in the two groups was the same.

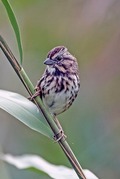

## 1 INTRODUCTION

Many organisms, especially outside of the tropics, utilize seasonal changes in day length (photoperiod) as a cue to modify physiological processes and to initiate or curtail behaviors in accord with seasonal conditions (Walton, Weil, & Nelson, 2011). Thus, investigating the effect of photoperiod on a particular behavior can provide insight into the seasonal function and phenology of that behavior. For example, aggressive behavior in a rodent species that typically breeds from April to September was experimentally increased by short photoperiods, perhaps because during the winter when shorter photoperiods occur it is advantageous to more aggressively compete for limited food resources (Jasnow, Huhman, Bartness, & Demas, 2000). Bird song is another behavior mediated by photoperiod (Smith, Brenowitz, & Wingfield, 1997). Because avian singing can be detected readily and passively in natural environments, there exists a unique opportunity to gain insight about how this behavior varies in response to photoperiod.

Bird song is known to function both in attracting mates and in defending territories in many species (Catchpole & Slater, 2008; Nowicki & Searcy, 2004). For example, muting caused red‐winged Blackbirds (*Ageliaus phoeniceus*) to lose their respective territories and then the recovery of the ability to sing resulted in territory reacquisition (Smith, 1979). Further, the quality of song displayed can affect the response of neighbors, with “high performance” songs less likely to stimulate an approach from neighbors (de Kort, Eldermire, Cramer, & Vehrencamp, 2009). Not only have females been shown to preferentially enter nest boxes from which male song was broadcast (Eriksson & Wallin, 1986), but repertoire size (Potvin, Crawford, MacDougall‐Shackleton, & MacDougall‐Shackleton, 2015; Reid et al., 2004) and “high performance” songs (Ballentine, 2004) have been shown to be attractive. Also, increased song sharing of song types with neighbors has been positively correlated with territory tenure in Song Sparrows (Beecher, Campbell, Burt, Hill, & Nordby, 2000). Though most year‐round, temperate species do not sing during the nonbreeding season, further supporting the breeding function of song, some species, like the Carolina Wren (*Thryothorus ludovicianus*), sing year‐round, which likely functions to defend food resources during the winter (Strain & Mumme, 1988).

Identifying both cues and mechanisms that mediate biologically important, and diverse, behaviors like song is a crucial first step toward predicting behavioral occurrence in particular conditions. It is well known that testosterone induces song in songbirds (Harding, 1988; Heid, Güttinger, & Pröve, 1985). In temperate‐breeding songbirds, photoperiod (specifically, increasing day length) stimulates the secretion of testosterone from gonads (Dawson, King, Bentley, & Ball, 2001) and enlarges vocal control regions in the brain which, in the presence of testosterone, results in singing behavior (Dloniak & Deviche, 2001). Though the interplay between photoperiod, testosterone, and singing behavior has been well studied, investigations of how migration and concomitant photoperiodic change affects singing behavior are lacking. One study (Kelsey, 1988) compared singing behavior of a long‐distance migrant, the Marsh Warbler (*Acrocephalus palustris*), on the breeding and nonbreeding grounds. However, no study that we are aware of has investigated the singing behavior of individuals of a species simultaneously at a location where that species occurs year‐round and at a location where that species solely overwinters. Lymburner, Kelly, Hobson, MacDougall‐Shackleton, and MacDougall‐Shackleton's study (2016) suggests that migration distance is negatively correlated with androgen concentration upon arrival on the breeding ground in males, which could also affect singing behavior.

It is currently unknown how individuals of a partially migratory species that utilize different strategies (latitudinal migration and nonmigration) compare with respect to singing behavior on the nonbreeding grounds when allopatric and then later on the breeding grounds when sympatric. Further, a comparison of singing behavior in both groups could be correlated with the ability of the focal species to respond to changes in climate. For example, species with greater variance in timing of breeding and associated behaviors will likely be better able to withstand environmental change (Fudickar & Ketterson, 2018). Generally, studies focusing on species during the nonbreeding season have been neglected (Marra, Cohen, Loss, Rutter, & Tonra, 2015), which may have resulted in nonbreeding functions of song being overlooked. In order to address both nonbreeding and breeding functions of song in birds and to understand variability in use and proximate mechanisms of song, there is a need to conduct controlled experiments rather than merely observational studies (Kroodsma & Byers, 1991).

We chose to address these needs and to study the partially migratory Eastern Song Sparrow (*Melospiza melodia melodia*). Several different subspecies of Song Sparrows are distributed throughout much of North America, particularly in riparian habitats, in both urban and rural environments. Song Sparrows breed as far north as 58°N and as far south as 19°N. Throughout most of their range north of 43°N, breeding individuals are migratory and overwinter to the south, just as individuals overwintering south of 33°N are migratory and breed to the north. Between 33°N and 43°N, some Eastern Song Sparrows are year‐round residents and some are migrants. Thus, during the nonbreeding season, Eastern Song Sparrows from the same breeding population may simultaneously experience a variety of photoperiods due to migration.

In this study, we sought to determine how inducing “migration” upon a group of captive male Eastern Song Sparrows in a laboratory setting affected timing and frequency of song output. We compared song output, which is correlated with mate attraction (MacDougall‐Shackleton, Stewart, Potvin, & Tennenhouse, 2009), in this group to another that we maintained at the photoperiod of both groups' breeding location (near Bloomington, Indiana). The simulated migration event put the migrant group on the same photoperiodic schedule as is experienced in Tampa, Florida, which is about 1,300 km south of Bloomington at the southern part of the nonbreeding range of Song Sparrows. We measured song output in both aviaries from November 2018 to April 2019. We induced “migration” again for the migratory group in mid‐March, such that by the end of the study both groups were experiencing the same photoperiod (as experienced in Bloomington, Indiana). We predicted that if photoperiod during the winter and early spring determine when and how many individuals sing, then there would be a difference in the onset and frequency of singing between the migrants and residents.

## 2 METHODS

### 2.1 Bird capture

Between 26 July and 22 August 2018, we captured 24 after‐hatch‐year Song Sparrows within a 1.1 km radius at a rural, agricultural site about 6 km east of Bloomington, Indiana. All birds captured were breeders at the site (or attempted breeders). We sexed each bird as male in the field by identifying the presence of a cloacal protuberance and by the bird's singing behavior. All birds were lured into mist nets with recordings of Song Sparrow song and then banded with a USGS metal band. A small quantity of blood (<50 µl) was collected from the brachial vein by venipuncture for molecular sex determination. We used quality of streaking of the breast and eye color to determine that our captured birds had already surpassed their hatch‐year (Pyle, 1997).

### 2.2 Photoperiod treatment and bird care

We randomly assigned all 24 birds to one of two, indoor aviaries maintained at 21°C, such that there were 12 birds in each aviary. The aviaries were similar in size (3.9 × 3.5 × 2.5 m and 3.5 × 3.1 × 2.5 m). Due to their year‐round territoriality, male Song Sparrows should be housed individually to avoid fighting (Smith, Hallager, Kendrick, Hope, & Danner, 2018). Thus, we individually housed each bird in a cage (0.6 × 0.6 × 0.5 m) and arranged four stacks of three cages, with each stack spaced 1.8 m from the stack across from it to standardize cage spacing in the aviary. We covered the back and one side of each cage with brown butcher paper so that each bird was only able to see birds across the aviary but not the birds in adjacent cages. We placed two perches in each cage, and birds were provided fresh food every two to three days, an ad libitum diet consisting of a mix of 2/3 white millet and 1/3 sunflower seed hearts, topped with eight mealworms, an orange slice, and a tablespoon of soft food (blended puppy chow, hard‐boiled egg, and carrots). In November, we began providing Nekton‐S Vitamin Supplement for Birds (Nekton GmbH, Germany) mixed in the Lanyon each time that we replaced food and water.

**Figure 1:**
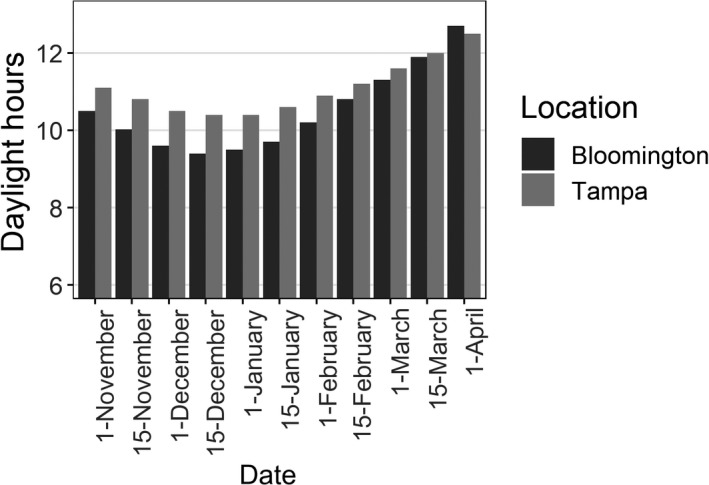
Daylight duration in Tampa, Florida (migrant treatment), and Bloomington, Indiana (resident treatment), during the duration of our study. Note the pronounced rate of change for Bloomington daylight compared to Tampa between 1 January and 1 April

Every two to three days, we adjusted automatic timers to shift the photoperiod in each aviary in accord with natural photoperiods in Bloomington, IN (39°N, 86°W; Figure 1). On 22 October, over a period of 5 days, we shifted one of the aviaries to the natural photoperiod of Tampa, FL (28°N, 82°W; Figure 1), which simulated a fall migration event. This migration duration is similar to how long Savannah Sparrows (*Passerculus sandwichensis)* would take to travel 1,300 km, according to one estimate (Mitchell, Woodworth, Taylor, & Norris, 2015). We considered these birds “migrants,” and refer to them as such hereafter. On 18 March, over a period of 5 days, we shifted the photoperiod of the aviary containing the migrants back to Bloomington, IN conditions, which simulated a spring migration event. The other aviary remained on a Bloomington, IN photoperiod for the duration of the study. We considered these birds “residents,” and refer to them as such hereafter.

### 2.3 Song recording and repertoire compilation

We used a camera (Sosun HD 1080 P 24.0MP) mounted on a tripod to record singing behavior in both aviaries every week between 1 November 2018 and 4 April 2019, with a recording session simultaneously occurring in both aviaries, on two (generally successive) days per week. We manually turned on cameras 10–15 min after lights were automatically turned on in a given aviary at “sunrise.” The camera recorded audio and video until memory ran out, which generally occurred after about 3.5 hr of operation. The movements of singing birds were sufficient to match individual birds and their song types, despite multiple birds sometimes singing simultaneously. To ensure that the birds in each aviary experienced auditory stimulus, every recording session we exposed the birds to playback of Song Sparrow song. In both aviaries, we placed a playback system (mp3 player: Ruizu X26; speaker: TDK Trek Micro A12) which was started each recording session at the same time that the camera was turned on. This system broadcast between 30 s and 1 min 15 s of song approximately every 30 min (same recording for both aviaries), beginning 5 min after the camera was turned on, and had a duration of 2 hr. The recordings consisted of songs from 3 Song Sparrows chosen randomly. The songs were recorded 6 km from the site that we captured the birds for the current experiment.

We determined song type repertoires for each individual so that instances of song that we recorded could be attributed to individuals, even when we did not see them singing on camera. Cassidy (1993) found that continuously recording 225 instances of Song Sparrow song is sufficient to capture the entire song repertoire, 4–13 song types, about 99% of the time (Cassidy, 1993). For one bird, a resident, we identified 303 instances of song and 9 song types. Because of the time‐intensive nature of identifying this many instances of song due to simultaneous singing in the rooms, we then set 75 song instances of song per bird as the minimum threshold for acquiring a repertoire of song types for every other bird. We continued counting songs for a bout of singing past 75 instances when it was still obvious which bird was singing. Not including the resident outlier, we recorded a mean of 81.2 ± 11.3 (S.D.) instances of song for the residents and 83 ± 11.7 instances of song for the migrants. Six Song Sparrows recorded in the field at the site where we captured our birds had completed a mean of 66% of their song type repertoire after we recorded ~75 songs (D. Brewer, personal observation), so we assume that we captured most of, but not the entire, song type repertoires of our captive birds. We also assume that both aviaries were equally represented with respect to proportion of song types identified.

We used Audacity (version 2.2.2) to generate spectrograms for each song type, using recordings extracted from the video cameras. Song Sparrows in at least one population do not alter their song repertoire after the first spring of their second calendar year of life (Nordby, Campbell, & Beecher, 2002), and such crystallization is generally assumed to be common in Song Sparrow populations. We assumed that our captive, adult Song Sparrows also had crystallized repertoires and did not learn the song types of other birds also in captivity during the study.

### 2.4 Stages and song counting

**Figure 2:**
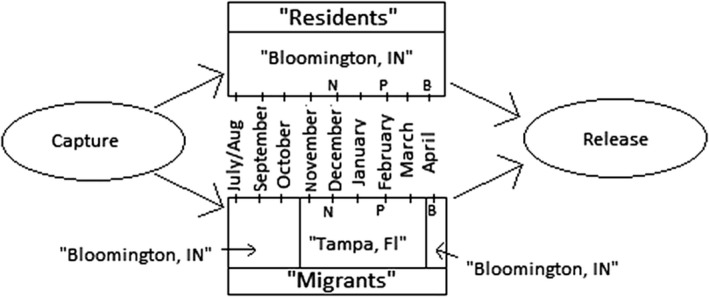
Schematic of events, from capture of birds through experiment to release of birds. In the “‘Migrants’ Box,” the vertical line between October and November indicates the 5‐day shift in photoperiod that simulated fall migration. In the same box, the vertical between March and April indicates the 5‐day shift in photoperiod that simulated spring migration. In both boxes, the N, P, and B denote “nonbreeding,” “prebreeding,” and “breeding.” These letters are centered between the two sampling dates that occurred during each stage (same dates for migrants and residents)

We defined three stages to describe breeding state, during which we counted the number of songs that individuals uttered. The dates defining the stages were based upon personal field observations of Song Sparrow behavior (D.B.) and based on the observations of Nice (1937), who observed Song Sparrows in Columbus Ohio (40°N, 83°W compared with Bloomington's 39°N, 86°W), and on observations of Arcese, Sogge, Marr, & Patten, 2002). The stages were “nonbreeding,” “prebreeding,” and “breeding,” and coincide with periods when breeding activity is not occurring, when territory establishment has begun among residents, and when mate attraction and nesting is occurring in the study area, respectively. We sampled two 30‐min periods in a given stage. Two weeks separated sampling dates in the prebreeding and breeding stages, and two weeks and two days separated sampling dates in the nonbreeding stage. Seven weeks separated the second sampling date in the nonbreeding stage and the first in the prebreeding stage. Six weeks separated the second sampling date in the prebreeding stage and the first in the breeding stage. The sampling dates for the stages were nonbreeding—20 November and 6 December; prebreeding—24 January and 7 February; breeding—21 March and 4 April. These events, along with capture, “migration,” and release dates, are overviewed in Figure 2.

For each 30‐min period analyzed (each began 30 min after the camera was turned on), we first generated spectrograms for all song types uttered and counted the number of times each song type was uttered. Then, we assigned the song types to individuals based upon the song types identified for individuals during the camera recording sessions. This allowed us to acquire a song count value for each individual. Though individuals did share some song types, subtle differences in song structure were generally sufficient to assign even shared song types to individuals, as in Beecher et al. (2000). When we were unsure what individual was associated with an instance of song, due to, for example, two birds having similar song types and masking by other singing birds covering identifiable elements of a particular instance of song, we disregarded that instance of song from analysis. We summed the two counts for each bird in a given stage and so acquired a single song count number for each bird in each stage in both aviaries. During each stage when singing primarily occurred (prebreeding and breeding), the proportion of song occurrences in each aviary not identified, and therefore not used in analysis, was similar (prebreeding: residents = 12.9%, migrants = 12.6%; breeding: residents = 19.1%, migrants = 19.7%).

### 2.5 Statistical analysis

We used generalized linear mixed models to analyze effects of resident status, breeding stage, and the interaction between resident status and breeding stage (fixed effects) on song output (our serial measurement), with individual identity as a random factor. Prior to the start of the experiment, one resident bird was excluded from sampling because its behavior appeared to be affected by an infection. Three additional birds were excluded from our analyses because they died in captivity from unknown illnesses, leaving 10 birds in each treatment. We only included the prebreeding and breeding stages in the analysis because the majority of birds in both treatments did not sing during the nonbreeding stage.

In order to normalize the distribution, we square root transformed song counts for each individual before the analysis. Statistical analysis was performed in SPSS vs 25 (IBM), and two‐tailed tests were used for all analyses, with a significance level of *p* = .05. All measures of variability are indicated by “±” standard error. Figures were made using R 3.6.0 (R Core Team, 2019).

### 2.6 Concurrent study

Once per week from February to March, we collected blood and measured fat from all birds in both aviaries on a day in which camera recordings did not take place. We assume that this affected both aviaries equally.

USFWS permit MB94788C‐0 approved collection of Song Sparrows from the wild, as did Indiana DNR permit 18‐049.

Captive protocols were approved by the Indiana University Institutional Animal Care and Use Committee # 18‐018.

## 3 RESULTS

For both migrants and residents, there was little song output during the nonbreeding stage (5 songs total for residents, three singers; 0 songs for migrants). During the prebreeding stage in both treatments, most birds sang and by the breeding stage all birds sang (Table 1).

**Table 1:** Overview of total song output by “resident” and “migrant” Song Sparrows in nonbreeding, prebreeding, and breeding stages, summed from two 30 min periods on different days in each stage

	Nonbreeding			Prebreeding			Breeding		
	Total songs (sang/didn't)	Mean # songs	Song range	Total songs (sang/didn't)	Mean # Songs	Song range	Total songs (sang/didn't)	Mean # Songs	Song range
Residents	3 (3/7)	0.5 ± 0.27	1–2	600 (10/ 0)	60 ± 14.68	2–237	542 (10/0)	54.2 ± 10.23	12–116
Migrants	0 (0/10)	0 ± 0	0 (all)	263 (8/ 2)	26.3 ± 12.14	0–115	535 (10/0)	53.5 ± 13.91	1–145

**Figure 3:**
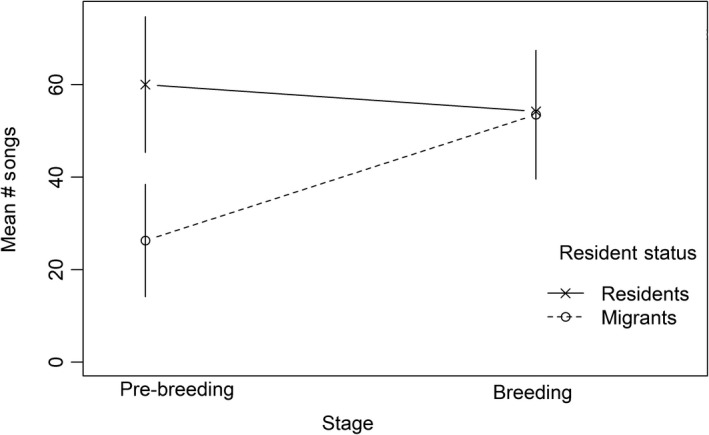
Resident Song Sparrows (x's) sing more than migrant Song Sparrows (open circles) during the prebreeding stage, but not during the breeding stage. Bars are 1 standard error.

**Figure 4:**
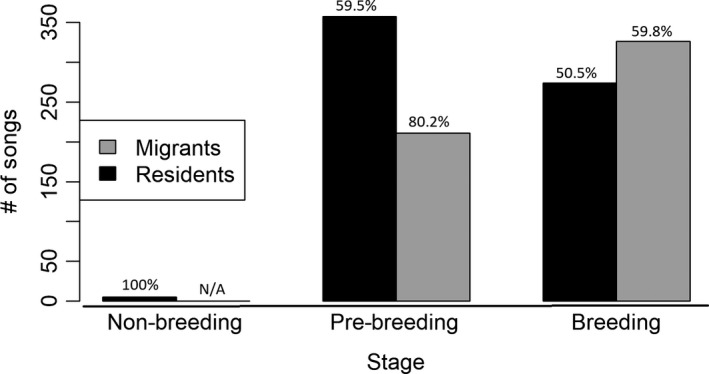
Sums of total number of songs for the three birds that sang the most in each stage for both migrants and residents. The percentage value above each bar indicates the percentage of all songs in a particular stage and group (migrants or residents) that the three birds collectively sang

Song output per individual did not vary based upon treatment alone (*F*_1, 18_ = 2.07, *p* = .167), though did tend to vary when breeding stage was considered (*F*_1, 18_ = 4.36, *p* = .051). There was an interaction between breeding stage and treatment (*F*_1, 18_ = 4.40, *p* = .050), with migrants and residents differing in song output during the prebreeding stage and not the breeding stage (Figure 3). In each group and stage, the three birds that sang the most uttered a disproportionate amount of the total songs, though that proportion became smaller for both migrants and residents as the stages progressed (Figure 4).

Light levels were most different between the treatments on 15 December, when photoperiod was one hour longer in Florida than in Bloomington, but by the beginning of April (end of study) Bloomington days had surpassed Tampa photoperiod length (Figure 1).

## 4 DISCUSSION

We investigated the effects of photoperiod associated with two overwintering locations on the seasonal onset of singing in a migratory songbird. Our results suggest that photoperiod during the late winter and early spring affects singing behavior in Eastern Song Sparrows, which was expected and is consistent with previous studies investigating the effects of photoperiod on singing behavior (Dloniak & Deviche, 2001; Smith et al., 1997). The novel finding of this study is that in our partially migratory study species, singing behavior varied between the breeding ground and the nonbreeding ground. This variance occurred during the period that we considered the prebreeding stage for the individuals overwintering on the breeding ground (late January through early February), but not during the beginning of the breeding stage. As expected, neither group sang at high rates during the nonbreeding stage, though at least three of the ten residents sang whereas none of the migrants sang. Residents may benefit from singing to defend their breeding territories during the nonbreeding season. If so, then producing song, even if greatly reduced in frequency, could be advantageous for residents during the nonbreeding season.

We defined the three breeding stages based on behavior observed in Bloomington, or at a similar latitude (Nice, 1937). Therefore, an observer in the southern part of the overwintering range of Song Sparrows may not consider the dates 21 January and 7 February to be within the “prebreeding” stage there, that is, there may be no behavior changes suggesting that individual Song Sparrows are preparing to breed during that time at that latitude. Our results suggest that the seasonal shift in behaviors associated with reproduction in Song Sparrows in the northern part of the overwintering range may occur earlier than in Song Sparrows wintering at the southern part of the overwintering range. It is known that there are advantages for males of many species to arrive earlier to the breeding grounds to facilitate breeding success (Morbey & Ydenberg, 2001), and Song Sparrows are likely no exception (Lymburner et al., 2016). In Song Sparrows, song functions both to attract mates (Reid et al., 2004) and to establish/defend territories (Nowicki, Searcy, & Hughes, 1998). Upon arrival to the breeding grounds, being in a behavioral state that is conducive to mate attraction and territory establishment/defense (like singing mediates) is likely crucial. Given that song output has been shown to be attractive to females (MacDougall‐Shackleton et al., 2009; Wasserman & Cigliano, 1991), it would likely be advantageous for a male to produce songs at a higher rate than competitors. Overwintering latitude and its effect on singing behavior could have fitness consequences if by the breeding stage migrants are at a song output disadvantage compared with residents. Based upon our definition of breeding stage, however, the migrants in our study would not have been at a competitive disadvantage based on song output alone upon initiation of the breeding stage (Figure 3), assuming that they were present. However, the proportion of total songs uttered by the three birds that sang the most was relatively high in the migrant group compared with the resident group, even during the breeding stage, which suggests that a transition may still have been occurring in some of the migrants with respect to initiating breeding‐level song output (Figure 4).

Overall, the pattern that we observed is consistent with the “rank advantage hypothesis” (Morbey & Ydenberg, 2001), with residents producing high rates of song earlier compared with migrants in order to compete for territories against other males already at the breeding grounds during the prebreeding stage. Given that the migrants likely would not be establishing territories at that point in time, it follows that their song output would be lower. However, considering that mean song rate for migrants during the prebreeding period was over 25 (in just 1 hr of sampling) (Table 1), then perhaps singing at this point in time is functional on the nonbreeding grounds, rather than merely being evidence of a transition to a state that would be useful on the breeding grounds. By 21 March, during the 5‐day shift in photoperiod that simulated migration, the migrants were singing at a rate that was the same as the residents. To our knowledge, singing rates of migrants in the field have not been examined during migration. Given that the simulated migration in our study occurred near the vernal equinox and that the difference between photoperiods in Bloomington and Tampa was five minutes when the simulation began, this “migration” shift itself likely had little impact upon song output. Testosterone, however, has been shown to increase during migration (Covino, Jawor, Kelly, & Moore, 2017), which could increase song rates. Regardless, the migrants in our study were no longer at a song output disadvantage at the time that they would have been arriving to the breeding grounds. Though we do not know how rapidly song increase occurred between the prebreeding and breeding stage for the migrants, we assume that the increase in rate was gradual, like Kelsey (1988) found in Marsh Warblers (*Acrocephalus palustris*) on the nonbreeding grounds.

Our study uniquely demonstrates how a natural change in photoperiod may affect singing behavior at two locations within a species range. Even though the migrants were on a longer photoperiod following the fall migration and throughout the prebreeding stage (Figure 1), they sang less frequently, which suggests that cues other than absolute day length are important for seasonal shifts in behavior. The resident treatment experienced more rapid changes in photoperiod (Figure 1) and sang more during the prebreeding stage (Figure 3). Therefore, we hypothesize that photoperiodic rate of change is a primary cause for increased frequency, and perhaps onset, of singing behavior in Eastern Song Sparrows. For migrants, the apparent relationship between testosterone levels at nonbreeding sites and migration phenology (Tonra, Marra, & Holberton, 2011) suggests that song output, which is mediated by testosterone levels (Harding, 1988; Heid et al., 1985), could be a salient predictor of when individuals will migrate in the spring and begin breeding.

The song output variance that we observed may be more pronounced when comparing individuals at nonbreeding and breeding locations separated by more distance. It is possible that future studies that compare locations separated by more distance could identify photoperiods and photoperiod rates of change that, unlike in our study, cause the migrant group to sing less than the resident group during the beginning of the breeding stage. If so, this would put migrants at a song output disadvantage that has fitness consequences. This disadvantage would be compounded by the trend of increasingly warmer spring temperatures in temperate zones that can cause early breeding (Both & Visser, 2001; Dunn & Winkler, 1999), which would likely favor residents and facultative migrants of both sexes.

Climate change may be causing a decline in migratory bird species because of the tendency for migration to be mistimed with respect to resource availability on the breeding grounds (Jones & Cresswell, 2010). In Song Sparrows, breeding readiness relates to song output, with an increase in song output coinciding with a seasonal increase in circulating testosterone (Nowicki & Ball, 1989). Longitudinal studies which utilize autonomous recording units (ARUS), or other means of passive monitoring, could determine how a salient breeding behavior like song relates to local resource availability. Especially for partially migratory species, this method could be used to determine advantages that residents may have over migrants with respect to being ready to breed when conditions are conducive. Given that individual Song Sparrows can be identified by the unique characteristics of their songs (Beecher et al., 2000) and that individuals return to the same or nearby breeding territories from year to year (Nice, 1937), it is feasible that onset of singing behavior on the nonbreeding ground in this species could be correlated with arrival to the breeding ground if ARUs were appropriately deployed. This would require both breeding and nonbreeding territories to be identified, though both can be remarkably constant from year to year in a number of species (Jahn et al., 2009). This noninvasive monitoring method could indicate whether overwintering latitude affects when individuals commence breeding activity on the breeding grounds. Further, this method could help conservation biologists determine if species with larger overwintering ranges (i.e., lower connectivity) are more resilient to environmental change due to greater variability in species‐wide timing of breeding than are species with smaller overwintering ranges (i.e., higher connectivity). If so, this knowledge could be applied when making decisions about how to use limited conservation resources.

Given that our study was done in the laboratory, we do not know how applicable our results are to the way that migrant and resident Song Sparrows behave in the field with respect to song output. The high density of individuals (particularly males, without females) confined together could affect song output (Boseret, Carere, Ball, & Balthazart, 2006), and less dominant males might have sung less in the lab than they otherwise would have (DeWolfe & Baptista, 1995). If temperature affects the onset of singing behavior in Song Sparrows, then our residents kept at a constant, relatively high winter temperature may have begun singing at high rates in the laboratory sooner than they would have in the field where temperatures are typically lower in late winter and early spring. Nice's (1937) data suggest that singing on 24 January, the first sampled day in our prebreeding stage, generally occurs if temperatures are greater than ~6°C whereas the laboratory was kept at 21°C. Further, females may be more likely than males to migrate to the southern part of the nonbreeding range (Lymburner et al., 2016). Another limitation of our study is that individuals which are genetically migrants or residents, rather than merely simulated migrants or residents, may respond differently to photoperiodic changes than did the simulated migrants and residents in our study. For example, the disproportionate amount of singing by three birds in the migrant group (Figure 4) could be because those birds were genetically residents. Nevertheless, our results suggest that within‐population differences in overwintering latitude may contribute to differences in the seasonal occurrence of singing.

In order to build upon our incipient understanding of how the singing behavior of songbirds varies in the nonbreeding and breeding range, laboratory or field studies which better account for real‐world variables are required. Such studies could help to elucidate functional advantages or disadvantages of differential frequency and onset of singing behavior. From a conservation perspective, understanding the degree to which migratory songbirds vary in timing of breeding, for which song could possibly serve as a proxy, may prove useful.

## CONFLICT OF INTEREST

We do not have any competing interests.

## AUTHOR CONTRIBUTIONS

DB caught the birds, designed the experiment, matched songs and singers, analyzed data, and wrote the manuscript. AF designed the experiment, analyzed the data, and helped revise the manuscript. CM matched songs and singers and approved the final manuscript.

## Data Availability

All data are available as Data S1.
